# Ambient and Bedroom Heat in Relation to Sleep Health in a Marginalized Community That Is One of the Hottest in Los Angeles

**DOI:** 10.3390/ijerph22091391

**Published:** 2025-09-06

**Authors:** Hasibe Caballero-Gomez, Jill Johnston, Chandra L. Jackson, Lizette Romano, Lara J. Cushing

**Affiliations:** 1 Department of Environmental Health Sciences, Fielding School of Public Health, University of California, Los Angeles, CA 90095, USA; hasibegomez@g.ucla.edu (H.C.-G.); lizette.romano@gmail.com (L.R.); 2 Department of Environmental and Occupational Health, University of California, Irvine, Irvine, CA 92697, USA; jillj1@hs.uci.edu; 3 Epidemiology Branch, National Institute of Environmental Health Sciences, National Institutes of Health, Department of Health and Human Services, Research Triangle Park, NC 27709, USA; chandra.jackson@nih.gov; 4 Intramural Research Program, National Institute on Minority Health and Health Disparities, National Institutes of Health, Department of Health and Human Services, Bethesda, MD 20892, USA

**Keywords:** health disparities, temperature, climate change, environmental justice

## Abstract

The majority of Americans do not regularly get the recommended amount of sleep and sleep deficiencies disproportionately burden marginalized communities. We conducted a longitudinal cohort study measuring bedroom air temperature and humidity over three non-consecutive weeks (*N* = 19 participants; 409 observation nights) using HOBO loggers and sleep health using wrist-actigraphy and sleep diaries. Outdoor temperature and humidity were obtained from a nearby weather station. Linear mixed-effects regression models assessed relationships between temperature and sleep health metrics. Nighttime indoor apparent temperature ranged from 26 to 35 °C and averaged 5 °C higher than outdoors. On average, participants slept 6.7 h per night with 83% sleep efficiency. After adjustment, a 5 °C increase in indoor nighttime dry bulb temperature was associated with a 23 min reduction in mean total sleep time (β = −23.30 [−43.30, −3.45]) and mean onset latency increase of approximately 2 min (β = 1.85 [0.50, 6.65]). Nighttime heat waves were associated with a 4% reduction in mean sleep efficiency (β = −3.71 [−6.83, −0.66]) and an 11 min increase in onset latency (β = 11.32 [2.60, 20.75]). We found evidence that rising summertime temperatures reduced sleep health in a disproportionately impacted community, suggesting that climate change will worsen existing sleep health disparities.

## 1. Introduction

Less than half of Americans get the recommended 7–8 h of sleep per day [1]. Insufficient sleep can increase the risk of depression, dementia, hypertension, type 2 diabetes, stroke, cardiovascular disease, and premature mortality [1,2]. Racial, ethnic, and socioeconomic disparities in sleep quality and disorders [3] are widening [4] and hypothesized to contribute to disparate rates of chronic illness among marginalized populations [5]. For example, one study estimates that more than half of the disparity in cardiometabolic risk between Black and White American adults can be explained by less sleep and lower sleep efficiency [6].

Core body temperature serves as an important biological cue for sleep onset and propensity [7], and high ambient temperatures have been associated with sleep health [8]. A prior study estimated that a 1 °C increase in nighttime temperature could produce nearly nine million additional nights of insufficient sleep per month in the United States (US) [9]. Average nighttime temperatures in Los Angeles are projected to increase by 2.7–4.1 °C by 2100 [10], highlighting the need to consider impacts to sleep health in the region.

Latines account for 40% of the population in California and represent the largest ethnic group in the US; yet, this population remains underrepresented in sleep research [11]. An actigraphy-measured sleep study found Latines aged 45–84 years had an 80% higher odds of sleeping less than six hours, when compared with their White counterparts, after adjusting for age, sex, and region [3]. During extreme heat days, California neighborhoods with the highest percentages of Latine residents experience land surface temperature differentials two times greater than other neighborhoods in the southwestern US, and this effect is more pronounced at night [12]. These findings highlight the importance of evaluating the susceptibility of this population to rising temperatures.

Our community-engaged Neighborhood Impacts of Global Warming on Sleep Health Trends (NIGHT) study evaluated the impact of high temperatures on sleep duration and quality among a convenience sample of Latine adults living in a community disproportionately burdened by heat in Los Angeles (LA), California. We utilized both self-reported and objective measures of sleep obtained via sleep diaries and actigraphy. Unlike prior studies that primarily relied on outdoor temperatures as a proxy of exposure, we collected indoor bedroom temperature and humidity for each participant. We hypothesized that increases in indoor temperatures and extreme heat events would shorten sleep duration and worsen sleep quality.

## 2. Materials and Methods

### 2.1. Study Site

This study was conducted in the LA neighborhood of Pacoima, CA, USA, where over 85% of residents identify as Latine. Pacoima was historically deemed ‘hazardous’ for investment in historical federal redlining maps [13], contributing to present day disparities in land cover and environmental burden [14]. The neighborhood is among the hottest in LA as a result of local meteorology and historical land use development [15,16]. Approximately 61% of Pacoima’s land cover is impervious heat-trapping surfaces, compared with Los Angeles County’s average of 48% [16]. CalEnviroScreen, a statewide screening tool ranks Pacoima among the 5% most pollution burdened communities in California due to an abundance of cleanup sites and poor air quality [17].

### 2.2. Participant Recruitment and Study Design

We partnered with Pacoima Beautiful, a grassroots community-based organization in the northeast San Fernando Valley, to recruit 19 adults (Figure S1). Participants were eligible if they were 18 years or older, resided in Pacoima, were not pregnant, had no record of a sleep disorder and, due to its documented influence on sleep quality and health, did not perform shift work [18]. Pacoima Beautiful staff supported recruitment by assisting with outreach to prospective participants via social media channels and flier distribution. Fliers were also distributed at public institutions and local community-serving organizations. After screening for eligibility, bilingual research staff arranged a home visit, where study participants completed a baseline health, sleep, housing, demographic, and heat behaviors and perceptions questionnaire in the language of their preference (English or Spanish). During this initial visit staff also instructed participants on the use of the sleep diary and wrist actigraphy, conducted a walk through inside and outside of the home to assess housing characteristics related to thermal comfort, and installed a temperature and humidity sensor in the room in which participants normally slept. Participants were instructed to not move the sensor, to wear the actigraph continuously for 24 h a day on seven consecutive days, and to simultaneously complete a sleep diary daily, after which equipment and diaries were retrieved from their home by study staff. Sleep and temperature data collection was repeated for two additional non-consecutive weeks roughly one month apart between August and October 2023. The study period was selected to include both the warmest months, August and September, and the cooler month of October to achieve temperature variation.

### 2.3. Sleep Outcome Measures

Philip Respironics Actiwatch (Spectrum Plus, model number: 1101894) devices were programmed to collect activity data in 30 s epochs starting midday on the participant’s first day of data collection. Actigraphy is a validated method for measuring sleep parameters using a noninvasive accelerometer, even against polysomnography, the gold standard in sleep measurements [19]. Sleep outcome metrics were generated using Philips Actiware 6 software, which provides daily total sleep time (in hours), sleep efficiency (%), sleep onset latency (in minutes), and wake after sleep onset (WASO, in minutes). Total sleep time refers to time scored as sleep during the sleep interval (total time elapsed between sleep onset and end). Sleep efficiency is the total sleep time divided by the sleep interval, expressed as a percentage. WASO measures the time spent awake during the sleep interval. Onset latency is the time elapsed between the start of rest interval and the start of the sleep interval. The Philips Actiware 6 software gives auto-detected suggestions for the start time of the rest and sleep intervals which had high agreement with the times participants reported getting in to bed and going to sleep using the sleep diary. We elected to use the software-generated onset latency values because they were missing less often than sleep diary entries. Total sleep time, sleep efficiency, WASO and onset latency were treated as continuous outcomes. Daily sleep medication use and subjective sleep quality ratings on a 5-point Likert scale were recoded using sleep diaries. Self–reported sleep quality was dichotomized into good (‘very good or ‘good’) or poor (‘fair,’ ‘poor,’ and ‘very poor’ self-reported sleep).

### 2.4. Environmental Measurements

Temperature and relative humidity were recorded at five-minute intervals using a HOBO temperature and dew point logger. The HOBO logger was installed on a shelf or tables 3 to 6 feet above the ground in a low traffic area in the room where the participant slept, away from any external heat sources (e.g., heaters, lights, windows), vents, air conditioners, fans, or humidifiers. HOBO loggers were placed in the same location for each week of data collection. We calculated apparent temperature (AT)—a measure that combines air temperature and humidity to better reflect how hot it feels—using a previously established formula [20]. Daily outdoor temperature and humidity data were obtained from the National Oceanic and Atmospheric Administration Climate Data Online archive for the Van Nuys airport [21], the nearest weather station in the area with continuous hourly data available during the study period.

### 2.5. Exposure Definition

Mean nighttime indoor apparent and dry bulb temperatures were calculated for each participant observation day. Nighttime was defined as 19:00 to 7:00 the following day, which corresponds roughly to sunset and sunrise during the midpoint of the study period. Days with daily maximum and minimum outdoor AT above the historical (1981–2020) August–October 90th percentile were defined as a hot day or night, respectively. If these temperatures persisted for at least two consecutive days or nights, they were labeled as daytime or nighttime heat events. The 90th percentile was chosen due to its documented association with heat–health outcomes in previous research conducted in southern California [22].

### 2.6. Statistical Analysis

Actigraphy measures that were two standard deviations from sample means were flagged as possible outliers and cross-verified with sleep diaries. If the actigraph deviated by less than 15% from the participant’s sleep diary and if the participant’s sleep diary was not weakly correlated to actigraphy-derived sleep measurements overall during the study period, then the actigraphy-derived value was considered legitimate and retained. If the actigraph deviated from the sleep diary by more than 15% it was classified as a true outlier and excluded from further analyses. We excluded 11 (2.7%), 11 (2.7%), 10 (2.4%), and 13 (3.2%) observations of total sleep time, sleep efficiency, WASO, and onset latency as outliers, respectively.

To test the hypothesis that higher indoor temperatures are associated with lower sleep quantity and worse quality, we constructed separate generalized linear mixed effects regression models for each sleep metric with a random intercept for participant to account for correlation between repeated measures. Total sleep time, sleep efficiency, and WASO were modelled as continuous outcomes using linear models. We used zero-inflated gamma models for onset latency, which had a high frequency of zeroes. These include both a binary component modeled using a logistic mixed effect regression, and a gamma component that uses a linear mixed effect model for non-zero observations. We assessed self-reported sleep quality (poor vs. good) using binomial models.

Continuous average nighttime indoor apparent and dry bulb temperatures were examined as primary predictors of each of our outcomes in separate models. To test the hypothesis that extreme heat events reduce sleep quantity and quality, we also ran models with a single binary independent variable for the presence or absence of a hot day, hot night, daytime or nighttime heat event based on outdoor temperature. We compiled a list of time-varying and time-invariant demographic and behavioral predictors of sleep quality and health from the literature to include as potential covariates. We first calculated correlation coefficients between all potential covariates and sleep health metrics to eliminate highly correlated predictors and those that were not strongly correlated with sleep outcomes in our study. We then utilized likelihood ratio tests to evaluate the impact of all remaining covariates on model fit, followed by a backward predictor elimination method, in which variables were iteratively removed to improve model performance, using both the Akaike information criterion (AIC) and the Bayesian information criterion (BIC). The following variables derived from the baseline questionnaire were found to improve model fit: presence of a diagnosed chronic illness, presence of a mental health challenge, secondhand smoke exposure, sleep medication use (time varying), and weekday versus weekend (time varying). We defined the presence of a mental health challenge as a score ≥3 on either the Generalized Anxiety Disorder 2-item scale or Patient Health Questionnaire-2, both previously validated and brief measures of depression and anxiety [23].

Each model was tested for convergence, singularity, normality, and constant variance. We compared unadjusted models, models adjusted for time-varying predictors, and fully adjusted models to evaluate the sensitivity of the effect estimates. In a sensitivity analysis, we included one- and two-day lags of the outcome variable as an additional predictor variable in models of sleep efficiency and total sleep time. REDCap electronic data were utilized for data collection and management hosted at the University of California, Los Angeles [24,25]. All statistical analysis was conducted using R version 4.3.3.

## 3. Results

### 3.1. Participant Characteristics and Sleep Metrics

Participants ranged in age, with the majority falling between 25 and 34 (32%), 35 and 44 (16%), and 45 and 55 (32%) years old. Most reported annual household incomes below USD 50,000 and identified as Mexican and female (Table 1). Two participants completed only 15 of the 21 days of data collection, and 13 participants wore the actigraph for longer than requested, resulting in a total of 409 observation nights. Participants on average slept less than the national guidelines (6.7 h vs. 7 to 8 h), spent 42 min awake after sleep onset, and had an onset latency of 19 min (Table 1). Sleep efficiency averaged 83% and participants reported good and fair sleep quality in nearly equal proportions (Table 1).

### 3.2. Temperature

Nighttime indoor AT was on average 5 °C hotter than nighttime outdoor AT and reached up to 12 °C hotter (Table 2). By comparison, daytime indoor AT was, on average, 1 °C cooler than outdoor daytime AT (Table 2).More than half of observations exceeded Baniassadi and colleagues identified optimal bedroom temperatures of 20–25 °C among an elderly sample [26] and over two thirds of observations exceeded California’s proposed maximum safe indoor air temperature of 27.8 °C for residential dwelling units [27]. Outdoor AT exceeded the historical 90th percentile on 11 nights of the 81-night study period. There were three instances where nighttime temperatures were above the 90th percentile for at least two consecutive nights (Table 2). Indoor temperatures were weakly to moderately correlated with outdoor temperatures (Spearman correlation coefficients ranging from 0.27 to 0.61), with the weakest correlation between indoor and outdoor minimum AT (Figure S2).

### 3.3. Regression Models

**Figure 1:**
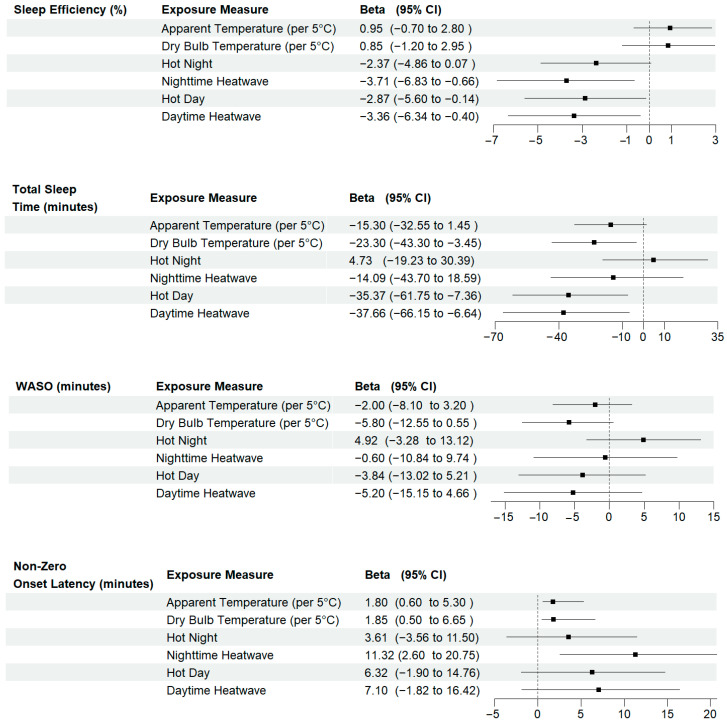
Mean differences and 95% confidence intervals for the association between a 5 °C increase in average nighttime indoor temperatures, extreme heat events, and sleep health metrics. Models control for chronic illness, anxiety/depression, secondhand smoke, weekday, and sleep medication use. CI = confidence interval. Dotted line represents the null.

A 5 °C increase in average nighttime dry bulb temperatures was associated with 23 min less total sleep time (β = −23.30 [−43.30, −3.45]), which was consistent across model specifications (Table S1). The effect estimate was attenuated when considering average AT (Figure 1). In the fully adjusted model, average total sleep times drop below the recommended seven hours when nighttime indoor dry bulb temperatures reach 24 °C. We also found significant reductions in total sleep time during hot days and daytime heat events (β = −35.37 [−61.75, −7.36] and β = −37.66 [−66.15, −6.64]) but less of an association with nighttime temperatures (Figure 1).

We observed <1% increases in sleep efficiency per 5 °C increase in average nighttime apparent and dry bulb temperature, but effect estimates crossed the null (Figure 1). Hot nights with minimum AT above the historical 90th percentile were associated with a 2% decrease in mean sleep efficiency (β = −2.37 [−4.86, 0.07]) that became stronger when the threshold was exceeded for two or more nights (β = −3.71 [−6.83, −0.66]) (Figure 1). Results were similar for hot days, with maximum AT above the historical 90th percentile and daytime heat events (Figure 1).

**Figure 2:**
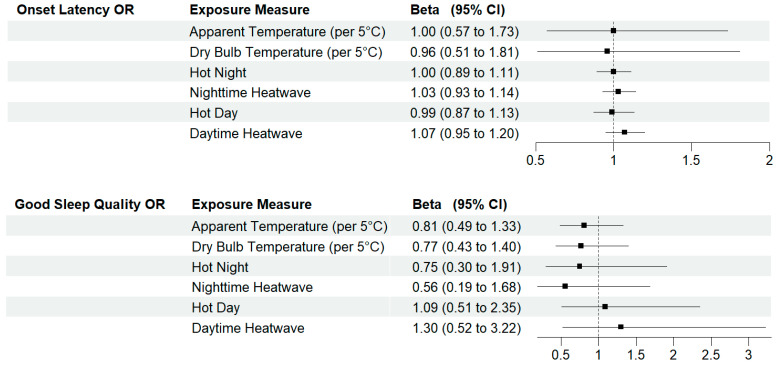
Odds ratios and 95% confidence intervals for the association between a 5 °C increase in average nighttime indoor temperatures, extreme heat events and sleep health metrics. Models control for chronic illness, anxiety/depression, secondhand smoke, weekday, and sleep medication use. CI = confidence interval; OR = odds ratio. Dotted line represents the null.

A 5 °C Celsius increase in average nighttime indoor apparent and dry bulb temperature increased non-zero onset latency by nearly two minutes ((β = 1.80 [0.60, 5.30]) and (β = 1.85 [0.50, 6.65]), respectively) (Figure 1). Both hot days and nights were also associated with increases in non-zero onset latency, with an 11 min increase with nighttime heat events (β = 11.32 [2.60, 20.75]). Associations between temperature, heat wave measures, and the odds of non-zero onset latency were null (Figure 2).

We found little evidence of an association between WASO and temperature, hot days, hot nights, or heat events (Figure 1). Higher nighttime temperatures were associated with lower odds of self-reported high-quality sleep, but confidence intervals were wide and crossed the null (Figure 2).

### 3.4. Sensitivity Analyses

Adding lagged outcome variables to our models of total sleep time and sleep efficiency slightly attenuated effect estimates and did not improve model fit (Tables S2 and S3). When using average outdoor nighttime apparent and dry bulb temperatures instead of indoor measurements, effect estimates were generally weaker, with wider confidence intervals (Table S4). The odds of a non-zero onset latency was higher when using outdoor rather than indoor temperature measures, although confidence intervals still included the null [AT: β = 1.20 (0.73, 1.96); dry bulb: β = 1.43 (0.81, 2.51)] (Table S4).

## 4. Discussion

Neighborhood environmental factors, such as noise, air quality, walkability, and access to natural amenities, have been shown to affect sleep and contribute to sleep health disparities [28]. Building on existing studies of temperature, we found a negative association between elevated indoor temperatures, extreme heat events and sleep quantity and quality in an understudied, predominately low income, Latine population. We observed elevated indoor warm season nighttime temperatures that regularly exceeded previously identified maximum temperatures for sleep health [26]. Higher indoor temperatures and unusually hot outdoor temperatures were associated with reductions in sleep duration and increases in non-zero onset latency. We also observed reductions in sleep efficiency with unusually hot outdoor temperatures. Two or more consecutive days or nights with unusually hot temperatures were more strongly associated with sleep than lone hot days or nights, suggesting greater impacts with more prolonged extreme heat events.

There was some variation in our effect estimates with respect to nighttime and daytime heat events, which often, but not always, overlapped. For example, both night- and daytime heat events were associated with reductions in sleep efficiency, while only daytime heat events were associated with reductions in total sleep time. This could arise—for example—if extreme daytime temperatures resulted in delayed bedtimes among individuals who needed to wake at a set time dictated by their work schedules or family responsibilities. The lack of association between total sleep time and nighttime heat events may also reflect the fact that our heat event measures were based on outdoor temperatures and did not reflect the use of indoor cooling systems, which may have been more prevalent at night when participants were more likely to be at home. Indoor temperatures above 24 °C corresponded to mean sleep durations less than the recommended seven hours of sleep in our sample. This is similar to the threshold of 25 °C identified in a previous study among an elderly sample [26]. Previous research among low-income, elderly adults also found elevated heart rates once temperatures exceeded 24 °C [29], a physiological sign associated with poorer sleep health [30].

Daily minimum temperatures are projected to increase by 2.7–4.1 °C in the Los Angeles area by 2100 [10]. Since the 1970s, Los Angeles has experienced an increased incidence of heat events lasting longer than six days [31], and heat events are projected to increase in length and frequency in the future [10]. Our results suggest that rates of chronic illness may accompany these temperature increases via poorer sleep, and that interventions to stabilize indoor temperatures are needed, particularly among historically marginalized communities that face higher risks of exposure and sleep deficiencies [12,15]. Lower sleep efficiency has been associated with depression [32], diabetes [33], and mortality [34]. A prior study of middle-aged adults found that sleeping less than seven hours a night was associated with a higher risk of hypertension, kidney disease, diabetes, high blood sugar, and multimorbidity [35]. Longer onset latencies in youth were found to be associated with chronic illness in the long term [36]. A health disparity of relevance to our study population is type 1 and type 2 diabetes, as US Latine populations disproportionately experience higher rates of morbidity and mortality compared with White populations, regardless of educational attainment or gender [37]. A study following over 15,000 Latines found those with short sleep durations and insomnia were 46% more likely to have diabetes compared with those who got the recommended amount of sleep and had no insomnia [38]. In our sample, participants slept under seven hours for over half (57%) of observation nights. These trends highlight a potential pathway for diabetes disparities among Latine populations that warrants further evaluation as temperatures continue to rise.

Our study was limited by a small sample size, which reduced our statistical power to detect associations between temperature and measures of sleep health. As our community-engaged study relied on convenience sampling, it is possible that people with undiagnosed sleep disorders or deficiencies were more likely to self-select into the study, leading us to overestimate the exposure effect. Additionally, although actigraphy demonstrates good validity in measuring sleep parameters, prior studies have shown low specificity in detecting wakefulness within sleep periods, which may explain our null findings regarding WASO [19]. We also did not consider cold temperature extremes in our study, which are rare in our study area but which have been associated with poorer sleep health in other studies. Further research with a larger and more representative sample could help confirm our findings and further characterize the relationship between temperature extremes and sleep health in other climates.

Strengths of our study include the inclusion of a largely understudied population and high degree of participant adherence. Our use of actigraphy as an objective measure of sleep health and collection of detailed indoor temperature measurements as well as individual-level covariates such as use of sleep aids and stress were additional strengths.

Several of our findings hold implications for future work. Outdoor temperatures served as an imperfect proxy for indoor temperature measurements and, on average, nighttime indoor temperatures were higher than nighttime outdoor temperature measurements in our study. When utilizing measures of continuous outdoor rather than indoor temperature, our effect estimates became attenuated and non-significant. This suggests that relying on outdoor temperature may miss some effects of temperature on sleep due to misclassification of exposure. Effect estimates related to self-reported sleep quality, although generally in the expected direction, were not as strong as the associations we observed using objective measures of sleep derived from actigraphy. Future studies should prioritize the collection of objective sleep and bedroom environmental measurements and include diverse study participants in order to further elucidate impacts on racially and ethnically minoritized populations.

## 5. Conclusions

In this longitudinal study of Latine adults from Los Angeles, CA, we found that elevated bedroom temperatures and unusually hot days and nights were associated with reductions in measures of sleep quantity and quality that have, in turn, been associated with poor mental and physical health [32,33,34,35,36,38], as well as premature mortality [2]. Our results suggest rising temperatures will worsen sleep health and may contribute to disparities in chronic illness that disproportionately impact marginalized communities.

## Figures and Tables

**Table 1:** Characteristics of the study population (*N* = 19 participants, 409 observation nights).

Study Population Characteristics	*N* (%)
Age in Years *N*(%)	
18–24	2 (11%)
25–34	6 (32%)
35–44	3 (16%)
45–55	6 (32%)
55+	2 (11%)
Sex, *N*(%)	
Female	17 (89%)
Male	2 (11%)
Educational Attainment, *N*(%)	
Less than high school	4 (21%)
High school or some college	7 (37%)
College degree	6 (32%)
Graduate degree	2 (11%)
Annual Household Income, *N*(%)	
Less than USD 10,000	5 (26%)
USD 10,000–USD 50,000	8 (42%)
More than USD 50,000	5 (26%)
Missing	1 (5%)
Ethnicity, *N*(%)	
Mexican	14 (74%)
Central or South American	4 (21%)
Filipino/a	1 (5%)
Mental Health Challenges, *N*(%)	
Yes	6 (32%)
No	13 (68%)
Chronic Illness, *N*(%)	
Yes	6 (32%)
No	13 (68%)
Housing Type, *N*(%)	
Single-family	8 (42%)
Multi-family	4 (21%)
Accessory dwelling unit	6 (32%)
Mobile home	1 (5%)
Bedroom Floor, *N*(%)	
First	15 (79%)
Second	4 (21%)
Bedroom Air Conditioning, *N*(%)	
Yes	13 (68%)
No	6 (32%)
Sleep efficiency (%), mean ± SD (range)	83 ± 9 (39–100)
Total sleep time (hours), mean ± SD (range)	6.7 ± 1.5 (3.4–13.6)
WASO (minutes), mean ± SD (range)	42 ± 25 (0–188)
Onset latency (minutes), mean ± SD (range)	19 ± 24 (0–160)
Self-Reported Sleep Quality, *N*(%) nights	
Very good	51 (13%)
Good	139 (34%)
Fair	148 (36%)
Poor	41 (10%)
Very poor	0
Missing	30 (7%)

SD = standard deviation.

**Table 2:** Heat exposure metrics. Nighttime was defined as 19:00 to 07:00 and daytime as 07:00 to 19:00.

Mean Nighttime Apparent Temperature (°C)		Mean (Range)
	Indoor	26 (20–35)
	Outdoor	22 (14–27)
	Indoor–outdoor difference	5 (−5–12)
Mean Nighttime Dry Bulb Temperature (°C)		Mean (Range)
	Indoor	27 (22–35)
	Outdoor	21 (15–28)
	Indoor–outdoor difference	5 (−5–13)
Mean Daytime Apparent Bulb Temperature (°C)		Mean (Range)
	Indoor	27 (20–35)
	Outdoor	28 (20–35)
	Indoor–outdoor difference	−1 (−12–7)
Mean Daytime Dry Bulb Temperature (°C)		Mean (Range)
	Indoor	26 (21–33)
	Outdoor	28 (20–36)
	Indoor–outdoor difference	−1 (−11–5)
Maximum Apparent Temperatures > 90th percentile (37.1 °C)		N (Observation Days)
	Hot days	9 (47)
	Daytime heat events	3 (40)
Minimum Apparent Temperatures > 90th percentile (22.8 °C)		N (Observation Days)
	Hot nights	11 (58)
	Nighttime heat events	3 (32)

## Data Availability

De-identified datasets generated during and/or analyzed during the current study are available upon reasonable request of the corresponding author.

## Supplementary Materials

The following supporting information can be downloaded at https://www.mdpi.com/article/10.3390/ijerph22091391/s1, Figure S1. Flow diagram of individuals at each stage of study; Table S1. Effect estimates and 95% confidence intervals for the association between a 5 °C increase in average nighttime indoor temperatures with sleep health metrics across model specifications; Table S2. Effect estimates and 95% confidence intervals for the association between a 5 °C increase in average nighttime indoor temperatures and sleep health metrics with sleep efficiency lags; Table S3. Effect estimates and 95% confidence intervals for the association between a 5 °C increase in average nighttime indoor temperatures and sleep health metrics with total sleep time lags; Table S4. Effect estimates and 95% confidence intervals for the association between a 5 °C increase in average nighttime outdoor temperatures and sleep health metrics; Figure S2. Spearman correlation coefficient plot for indoor and outdoor apparent (top) and dry bulb (bottom) temperature measurements.
