# Supplementary material for: Ambient and Bedroom Heat in Relation to Sleep Health in a Marginalized Community That Is One of the Hottest in Los Angeles
Source: Int J Environ Res Public Health. 2025 Sep 6;22(9):1391. doi: 10.3390/ijerph22091391 (PMC12469264; doi:10.3390/ijerph22091391)
Supplement: Supplementary file 1 [file ijerph-22-01391-s001.zip › ijerph-3804111-supplementary.pdf]

Supplementary Information

Ambient and Bedroom Heat in Relation to Sleep Health in a Marginalized Community that is One of the Hottest in Los Angeles

Figure S1. Flow Diagram of Individuals at Each Stage of Study 2

Table S1. Effect estimates and 95% confidence intervals for the association between a 5°C increase in average nighttime indoor temperatures with sleep health metrics across model specifications 3

Table S2. Effect estimates and 95% confidence intervals for the association between a 5°C increase in average nighttime indoor temperatures and sleep health metrics with sleep efficiency lags 4

Table S3. Effect estimates and 95% confidence intervals for the association between a 5°C increase in average nighttime indoor temperatures and sleep health metrics with total sleep time lags 5

Table S4. Effect estimates and 95% confidence intervals for the association between a 5°C increase in average nighttime outdoor temperatures and sleep health metrics 6

Figure S2. Spearman Correlation Coefficient plot for Indoor and Outdoor Apparent (top) and Dry Bulb (bottom)Temperature Measurements... 7

Figure S1. Flow Diagram of Individuals at Each Stage of Study

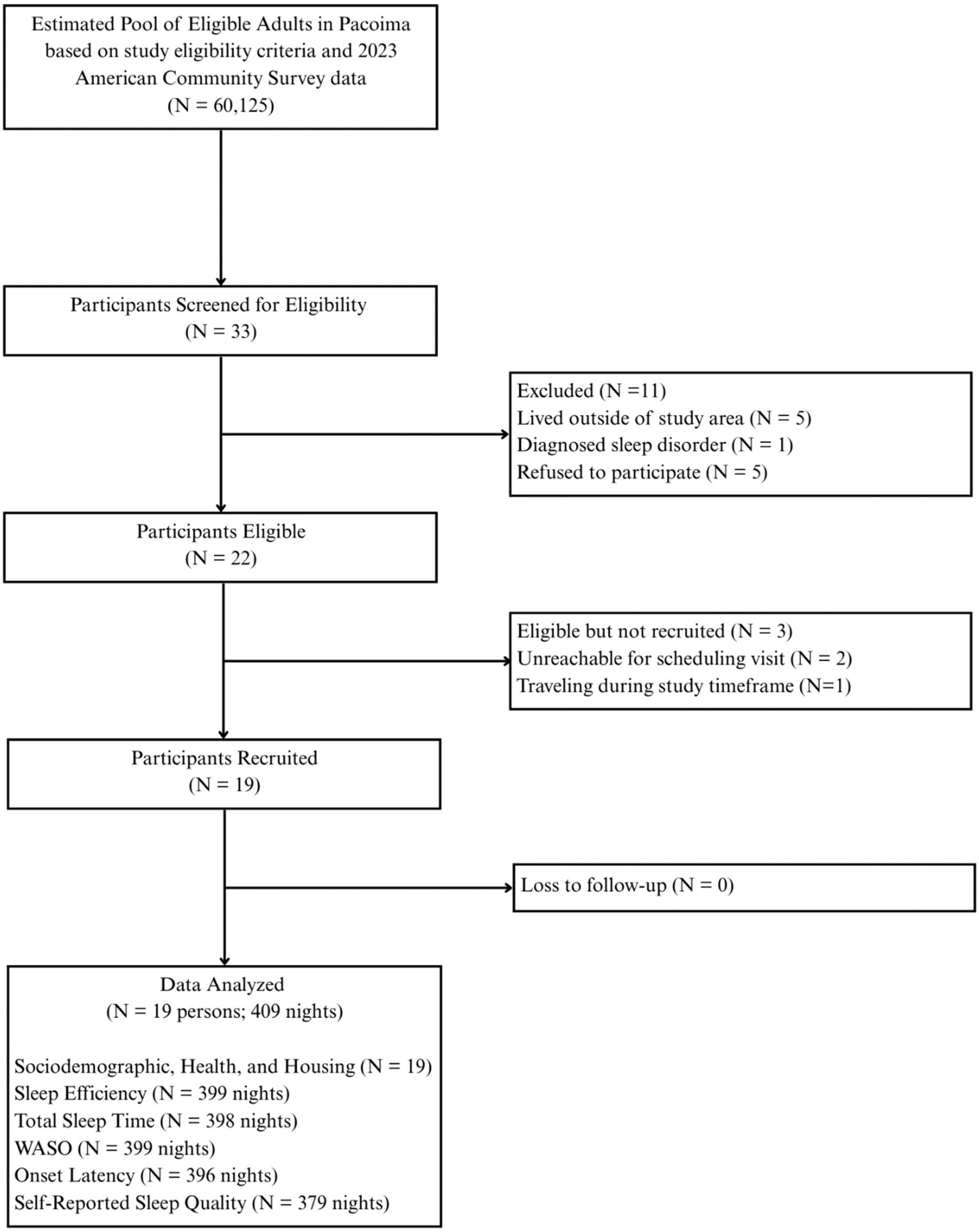

Table S1. Effect estimates and 95% confidence intervals for the association between a 5°C increase in average nighttime indoor temperatures with sleep health metrics across model specifications

Mean Difference (95% CI)						
	AT[1] Unadjusted	AT Model 1	AT Model 2	DB[2] Unadjusted	DB Model 1	DB Model 2
Sleep Efficiency (%)	1.05 (-0.70, 2.80)	1.05 (-0.65, 2.80)	0.95 (-0.70, 2.80)	0.50 (-1.55, 2.65)	0.90 (-1.15, 3.00)	0.85 (-1.20, 2.95)
Total Sleep Time (minutes)	-18.00 (-35.15,-0.65)	-15.75 (-33.10, 1.40)	-15.30 (-32.55, 1.45)	-23.50 (-44.20,-3.00)	-22.50 (-42.80,-2.25)	-23.30 (-43.30,-3.45)
WASO (minutes)	-2.80 (-8.10, 2.65)	-1.95 (-7.65, 3.60)	-2.00 (-8.10, 3.20)	-6.10 (-12.50, 0.20)	-5.80 (-12.45, 0.75)	-5.80 (-12.55, 0.55)
Non-Zero Onset Latency (minutes)	2.05 (0.70, 5.80)	1.90 (0.65, 5.75)	1.80 (0.60, 5.30)	2.10 (0.60, 7.45)	1.90 (0.55, 7.05)	1.85 (0.50, 6.65)
Odds Ratio (95% CI)						
	AT Unadjusted	AT Model 1	AT Model 2	DB Unadjusted	DB Model 1	DB Model 2
Onset Latency	1.08 (0.62, 1.88)	1.08 (0.63, 1.86)	1.00 (0.57, 1.73)	1.06 (0.54, 2.06)	1.03 (0.55, 1.96)	0.96 (0.51, 1.81)
Self-Reported Sleep Quality	0.81 (0.49, 1.34)	0.80 (0.48, 1.32)	0.81 (0.49, 1.33)	0.80 (0.44, 1.46)	0.78 (0.43, 1.41)	0.77 (0.43, 1.40)

[1]AT = Apparent Temperature [2]DB = Dry Bulb Temperature

Model 1: Adjusts for weekday vs weekend and sleep medication use

Model 2: Adjusts for chronic illness, anxiety/depression, secondhand smoke, weekday vs weekend, sleep medication use

Table S2. Effect estimates and 95% confidence intervals for the association between a 5°C increase in average nighttime indoor temperatures and sleep health metrics with sleep efficiency lags

	Mean Difference (95% CI)			
	AT[1] 1-day lag	AT 2-day lag	DB[2] 1-day lag	DB 2-day lag
Sleep Efficiency (%)	0.90 (-0.80, 2.75)	0.75 (-1.10, 2.65)	0.55 (-1.50, 2.65)	0.35 (-1.85, 2.50)
Total Sleep Time (minutes)	-16.00 (-33.30, 1.25)	-12.75 (-30.80, 5.50)	-19.80 (-40.05, 0.45)	-18.60 (-39.40, 2.55)
WASO (minutes)	-1.30 (-7.40, 3.80)	-0.50 (-6.75, 4.80)	-3.90 (-10.70, 2.35)	-3.40 (-10.30, 3.10)
Non-Zero Onset Latency (minutes)	1.80 (0.55, 5.60)	2.45 (0.75, 7.95)	2.10 (0.55, 8.10)	2.05 (0.55, 7.95)
	Odds Ratio (95% CI)			
	AT 1-day lag	AT 2-day lag	DB 1-day lag	DB 2-day lag
Onset Latency	0.90 (0.55, 1.47)	0.93 (0.55, 1.57)	0.96 (0.54, 1.73)	1.04 (0.56, 1.93)
Self-Reported Sleep Quality	0.80 (0.48, 1.33)	0.80 (0.47, 1.37)	0.79 (0.43, 1.46)	0.72 (0.38, 1.34)

[1]AT = Apparent Temperature [2]DB = Dry Bulb Temperature
Model controls for anxiety/depression, chronic illness, secondhand smoke, weekday vs weekend, sleep medication use, and prior nights sleep efficiency

Table S3. Effect estimates and 95% confidence intervals for the association between a 5°C increase in average nighttime indoor temperatures and sleep health metrics with total sleep time lags

	Mean Difference (95% CI)			
	AT[1] 1-day lag	AT 2-day lag	DB[2] 1-day lag	DB 2-day lag
Sleep Efficiency (%)	0.65<br>(-1.05, 2.55)	0.65<br>(-1.20, 2.65)	0.25<br>(-1.85, 4.50)	0.35<br>(-1.90, 2.65)
Total Sleep Time (minutes)	-14.35<br>(-31.05, 3.40)	-11.55<br>(-28.90, 7.65)	-16.85<br>(-36.55, 4.15)	-17.80<br>(-38.05, 4.90)
WASO (minutes)	0.00<br>(-5.65, 4.50)	0.35<br>(-5.50, 5.25)	-2.60<br>(-8.95, 3.05)	-2.80<br>(-9.25, 3.25)
Non-Zero Onset Latency (minutes)	1.95<br>(0.60, 6.30)	2.85<br>(0.85, 9.55)	2.05<br>(0.55, 8.10)	2.05<br>(0.50, 8.25)
	Odds Ratio (95% CI)			
	AT 1-day lag	AT 2-day lag	DB 1-day lag	DB 2-day lag
Onset Latency	0.88<br>(0.50, 1.56)	0.89<br>(0.53, 1.49)	0.91<br>(0.46, 1.79)	0.96<br>(0.51, 1.79)
Self-Reported Sleep Quality	0.80<br>(0.48, 1.34)	0.87<br>(0.50, 1.49)	0.76<br>(0.41, 1.40)	0.76<br>(0.40, 1.44)

[1]AT = Apparent Temperature [2]DB = Dry Bulb Temperature
Model controls for anxiety/depression, chronic illness, secondhand smoke, weekday vs weekend, sleep medication use, and prior nights total sleep time

Table S4. Effect estimates and 95% confidence intervals for the association between a 5°C increase in average nighttime outdoor temperatures and sleep health metrics

Mean Difference (95% CI)						
	AT[1] Unadjusted	AT Model 1	AT Model 2	DB[2] Unadjusted	DB Model 1	DB Model 2
Sleep Efficiency (%)	-0.30 (-1.80, 1.20)	-0.10 (-1.65, 1.40)	-0.10 (-1.65, 1.40)	-0.15 (-1.75, 1.45)	0.25 (-1.40, 1.85)	0.20 (-1.45, 1.85)
Total Sleep Time (minutes)	-13.00 (-29.65, 3.90)	-11.35 (-26.20, 3.85)	-14.60 (-29.65, 4.10)	-15.00 (-32.60, 3.00)	-14.95 (-31.10, 1.50)	-15.25 (-31.00, 1.65)
WASO (minutes)	0.20 (-4.30, 4.75)	0.25 (-4.65, 5.15)	0.25 (-4.60, 5.15)	-1.80 (-6.65, 3.05)	-2.55 (-7.85, 2.80)	-2.50 (-7.75, 2.90)
Non-Zero Onset Latency (minutes)	4.80 (-1.15, 10.90)	1.00 (-3.45, 5.65)	1.10 (-3.15, 5.95)	6.75 (0.40, 13.20)	2.10 (-2.80, 7.10)	2.00 (-2.70, 7.15)
Odds Ratio (95% CI)						
	AT Unadjusted	AT Model 1	AT Model 2	DB Unadjusted	DB Model 1	DB Model 2
Onset Latency	1.20 (0.75, 1.91)	1.17 (0.72, 1.92)	1.20 (0.73, 1.96)	1.48 (0.88, 2.50)	1.42 (0.81, 2.49)	1.43 (0.81, 2.51)
Self-Reported Sleep Quality	0.87 (0.57, 1.33)	0.86 (0.56, 1.31)	0.86 (0.57, 1.32)	0.89 (0.57, 1.40)	0.86 (0.55, 1.36)	0.87 (0.55, 1.37)

[1]AT = Apparent Temperature [2]DB = Dry Bulb Temperature

Model 1: Adjusts for weekday vs weekend and sleep medication use

Model 2: Adjusts for chronic illness, anxiety/depression, secondhand smoke, weekday vs weekend, sleep medication use

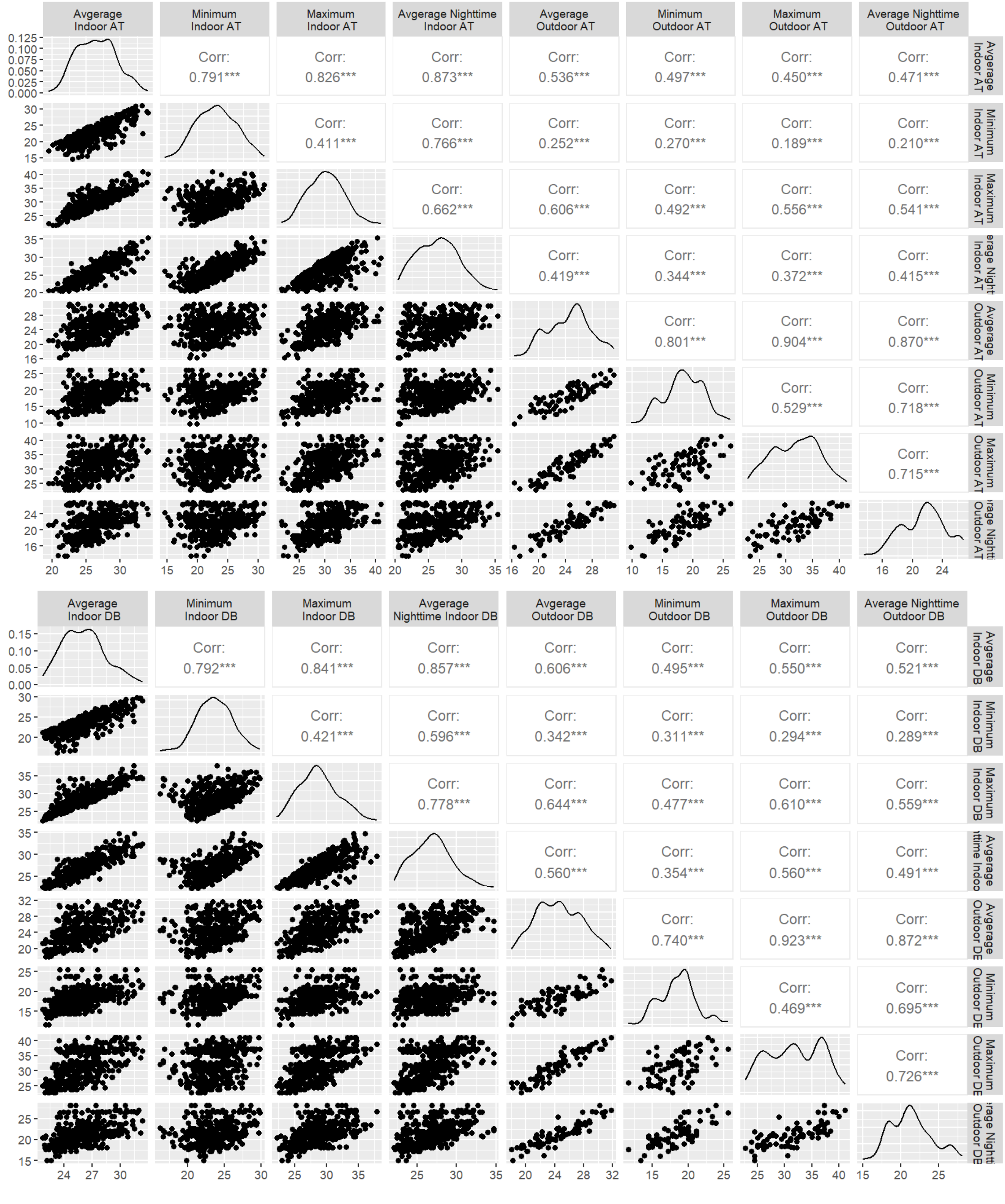

Figure S2. Spearman Correlation Coefficient plot for Indoor and Outdoor Apparent (top) and Dry Bulb (bottom)Temperature Measurements.

AT = Apparent Temperature, DB = Dry Bulb Temperature
